# Chronic Rhinosinusitis Is Associated with Lower Airway Mucus Plugging

**DOI:** 10.3390/jcm15197609

**Published:** 2026-09-30

**Authors:** Akane Masuda, Yuta Kono, Ryo Maruyama, Haruka Motohashi, Naoya Aoshiba, Mariko Kogami, Mayuko Ishiwari, Ryota Kikuchi, Kenichi Kobayashi, Kiyoaki Tsukahara, Shinji Abe

**Affiliations:** 1 Department of Respiratory Medicine, Tokyo Medical University, 6-7-1 Nishi-Shinjuku, Shinjuku-ku, Tokyo 160-0023, Japan; akane112358@gmail.com (A.M.); krhrtmthshrk@icloud.com (H.M.); aoshiba.708.smu@gmail.com (N.A.); kyokui110008@gmail.com (M.K.); mayuko_hojo@yahoo.co.jp (M.I.); reo1129@tokyo-med.ac.jp (R.K.); k-kobayashi@nms.ac.jp (K.K.); sabe@tokyo-med.ac.jp (S.A.); 2 Department of Otorhinolaryngology, Head and Neck Surgery, Tokyo Medical University, 6-7-1 Nishi-Shinjuku, Shinjuku-ku, Tokyo 160-0023, Japan; ryomaru@tokyo-med.ac.jp (R.M.); tsuka@tokyo-med.ac.jp (K.T.)

**Keywords:** chronic rhinosinusitis, mucus plugs, eosinophilic chronic rhinosinusitis, bronchiectasis, asthma

## Abstract

**Background:** Chronic rhinosinusitis (CRS) is associated with lower airway diseases, but its relationship with mucus plugging remains unclear. We investigated the association between CRS and lower airway mucus plugging. **Methods:** This single-center retrospective observational study included patients who underwent paranasal sinus computed tomography and chest high-resolution computed tomography. Associations between CRS and mucus plugging were evaluated, including phenotype-stratified analyses according to eosinophilic CRS (ECRS) status. **Results:** Among 97 patients, 51 had CRS and 46 did not. Mucus plugging was more frequent in patients with CRS than in those without CRS (41.2% vs. 10.9%, *p* = 0.001), and mucus plug scores were higher in the CRS group (*p* = 0.002). CRS remained independently associated with mucus plugging after adjustment for asthma and bronchiectasis (adjusted odds ratio, 4.06; 95% confidence interval, 1.30–12.7; *p* = 0.016). Mucus plugging was more prevalent in both the non-ECRS and ECRS groups than in the non-CRS group, with no significant difference between the two CRS phenotypes. **Conclusions:** CRS remained associated with mucus plugging after adjustment for asthma and bronchiectasis. Mucus plugging may represent an underrecognized radiological feature of the lower airways in CRS.

## 1. Introduction

The upper and lower airways constitute a continuous mucosal epithelial system and share common immunological and inflammatory pathways, thereby functioning as an integrated airway unit [1]. This concept, widely recognized as the united airway concept, proposes that inflammation arising in the upper airway can substantially influence lower airway pathophysiology [2]. Within this framework, chronic rhinosinusitis (CRS), a chronic inflammatory disorder of the sinonasal mucosa, has been consistently associated with lower airway diseases, most notably asthma and bronchiectasis [3,4].

A bidirectional association between CRS and asthma has been demonstrated in clinical studies. Patients with asthma have a significantly increased risk of developing CRS, while CRS is highly prevalent among individuals with asthma [5,6]. This association is particularly strong in patients with nasal polyps and is underpinned by shared type 2 inflammatory pathways. Moreover, a greater radiological burden of sinus disease has been reported to correlate with greater asthma severity [7].

CRS is also closely associated with bronchiectasis. Studies indicate that a substantial proportion of patients with bronchiectasis have coexisting CRS and, conversely, that radiographic bronchiectasis is frequently identified in patients with CRS [4,8]. Longitudinal observations suggest that CRS often precedes the diagnosis of bronchiectasis by several years, raising the possibility that upper airway inflammation contributes to lower airway structural damage [9]. CRS has also been associated with worse clinical outcomes in patients with bronchiectasis [4]. Taken together, these findings suggest that CRS is associated with both the presence and severity of asthma and bronchiectasis.

Growing attention has focused on lower airway mucus plugs as an important pathological feature of chronic airway diseases. In asthma, mucus plugs are prevalent, particularly in severe disease, and are strongly associated with airflow limitation, ventilation defects, symptom burden, and exacerbation risk [10,11,12]. These mucus plugs can persist over time and are pathophysiologically linked to type 2 inflammation [10,13]. Consistent with this, mucus plugging is more frequently observed in patients with asthma and nasal polyps, a phenotype strongly associated with type 2 inflammation [14]. Mucus plugs are also common in bronchiectasis and are associated with greater disease severity [15,16]. Sputum myeloperoxidase concentrations have been reported to correlate with the extent of mucus plugging, suggesting that neutrophilic inflammation contributes to mucus plug formation in bronchiectasis [16]. Collectively, these findings indicate that mucus plugging is clinically relevant to disease severity in both asthma and bronchiectasis.

CRS has been associated with greater severity of asthma and bronchiectasis, and mucus plugging is also associated with greater disease severity in these conditions. However, the relationship between CRS and lower airway mucus plugging remains insufficiently explored. From the perspective of the united airway concept, CRS may contribute to mucus plug formation in the lower airways. On this basis, we investigated whether CRS was associated with the presence and extent of lower airway mucus plugging and explored whether these associations differed according to the eosinophilic CRS (ECRS) phenotype.

## 2. Materials and Methods

### 2.1. Study Design and Participants

This was a single-center, retrospective observational study. We screened consecutive patients aged ≥16 years who visited the Department of Otolaryngology–Head and Neck Surgery at Tokyo Medical University Hospital between October 2022 and April 2026. Eligible patients had undergone paranasal sinus computed tomography (CT) and chest high-resolution computed tomography (HRCT) performed no earlier than 1 month before the paranasal sinus CT. A total of 176 patients were initially identified.

To minimize potential confounding of HRCT findings, patients with conditions that could independently cause bronchiectatic changes, including interstitial lung disease (*n* = 14), allergic bronchopulmonary mycosis (*n* = 1), nontuberculous mycobacterial disease (*n* = 2), primary ciliary dyskinesia (*n* = 1), or diffuse panbronchiolitis (*n* = 1), were excluded. Patients with conditions that precluded adequate whole-lung assessment, including prior lung resection (*n* = 9) or pleural effusion (*n* = 1), were also excluded. Conditions that could affect the assessment of CRS, including odontogenic maxillary sinusitis (*n* = 9), sinonasal tumors (*n* = 3), and fungal sinusitis (*n* = 1), were also excluded. Furthermore, patients receiving systemic corticosteroid therapy (*n* = 12) or biologic agents (*n* = 4), those with hematologic disorders (*n* = 16), active inflammatory conditions (pneumonia, *n* = 2; bacteremia, *n* = 1; chronic osteomyelitis, *n* = 1), or missing laboratory data (*n* = 1) were excluded because of their potential effects on peripheral blood inflammatory markers. After applying these exclusion criteria, 97 patients were included in the final analysis.

The date of paranasal sinus CT was defined as the index date. Clinical information, including age, smoking history, and comorbidities, was retrospectively collected from the medical records with reference to the index date. Asthma and allergic rhinitis were defined based on a physician-confirmed diagnosis documented in the medical record. For laboratory variables, data obtained closest to the index date during a clinically stable period were used. For chest imaging, the first chest HRCT performed no earlier than 1 month before the index date was used for evaluation. This temporal criterion was used to avoid inclusion of chest HRCT performed substantially before the sinonasal evaluation while retaining patients with pre-existing CRS symptoms shortly before sinus CT confirmation. Using these clinical, laboratory, and imaging data, the association between CRS and lower airway mucus plugging was retrospectively assessed. The principal analysis focused on the presence of lower airway mucus plugs on chest HRCT and its association with CRS. Mucus plug score was additionally evaluated as a secondary measure of mucus plug burden. Comparisons according to CRS phenotype (non-CRS, non-ECRS, and ECRS) were considered exploratory analyses.

The clinical indications for chest CT and paranasal sinus CT were retrospectively reviewed from the medical records. Indications for chest CT were categorized as respiratory symptoms, respiratory disease or abnormal chest findings, non-respiratory symptoms or abnormal findings, or evaluation, follow-up, or screening of other diseases. Indications for paranasal sinus CT were categorized as sinonasal symptom-related or other indications. Individual indications for both examinations are provided in Supplementary Table S1.

This study was approved by the Ethics Committee of Tokyo Medical University (approval number: T2025-0079). Given the retrospective nature of the study, the requirement for written informed consent was waived, and patients were provided with the opportunity to opt out of the study.

### 2.2. Definition and Assessment of Chronic Rhinosinusitis

CRS was defined as the presence of at least one sinonasal symptom (nasal obstruction, nasal discharge, olfactory dysfunction, or facial pain/pressure) persisting for ≥12 weeks, together with paranasal sinus CT findings compatible with CRS. Although nasal endoscopy was not required for CRS diagnosis, all included patients with CRS underwent nasal endoscopic examination to assess the presence of nasal polyps. Both unilateral and bilateral CRS were included. Patients with major secondary causes of unilateral sinonasal disease, including odontogenic maxillary sinusitis, fungal sinusitis, and sinonasal tumors, were excluded, as described above.

Eosinophilic CRS (ECRS) was defined using the Japanese Epidemiological Survey of Refractory Eosinophilic Chronic Rhinosinusitis (JESREC) scoring system [17]. The JESREC score was calculated based on four components: bilateral sinonasal lesions (3 points), presence of nasal polyps (2 points), ethmoid sinus–dominant disease on CT (2 points), and peripheral blood eosinophil percentage (>2% to ≤5%, 4 points; >5% to ≤10%, 8 points; and >10%, 10 points). For the purposes of this study, ECRS was clinically defined as a JESREC score of ≥11, irrespective of histopathological findings. Patients with CRS who did not meet the criteria for ECRS were classified as non-ECRS.

The severity of CRS was assessed using the Lund–Mackay (LM) scoring system based on paranasal sinus CT findings. Each sinus (maxillary, frontal, sphenoid, anterior ethmoid, and posterior ethmoid) was scored as 0 for no opacification, 1 for partial opacification, or 2 for complete opacification. The ostiomeatal complex was scored as 0 if not obstructed and 2 if obstructed. Total LM scores ranged from 0 to 24, with higher scores indicating more extensive sinonasal disease.

### 2.3. Definition and Assessment of Bronchiectasis

Bronchiectasis was diagnosed based on HRCT findings when at least one of the following criteria was met: (1) an internal bronchial diameter greater than that of the accompanying pulmonary artery, (2) lack of normal bronchial tapering toward the periphery, or (3) visualization of bronchi within 1 cm of the pleural surface. All HRCT scans were acquired at full inspiration with a slice thickness of 1.2 mm.

The radiological severity of bronchiectasis was assessed using the Bronchiectasis Radiologically Indexed CT Score (BRICS), an HRCT-based scoring system that combines the degree of bronchial dilatation and the number of bronchopulmonary segments with emphysema [18]. BRICS ranges from 0 to 5, with higher scores indicating greater radiological severity. Two pulmonologists (Y.K. and A.M.) independently reviewed the CT scans, and discrepancies were resolved by consensus.

### 2.4. Definition and Assessment of Lower Airway Mucus Plugs

Lower airway mucus plugs were identified on HRCT as round or branching opacities completely occluding the bronchial lumen. Complete airway obstruction attributable to intraluminal mucus was required for classification as a mucus plug. The mucus plug score was calculated as the number of bronchopulmonary segments containing at least one mucus plug and ranged from 0 to 18 [10,19]. Mucus plugs were assessed independently by two pulmonologists (Y.K. and A.M.) who were blinded to CRS status, with disagreements resolved by consensus.

### 2.5. Statistical Analysis

Continuous variables are presented as medians with interquartile ranges (IQR), and categorical variables as numbers and percentages. Univariable logistic regression analyses were performed to evaluate the association of each candidate factor with the presence of mucus plugs. The primary multivariable logistic regression model included CRS, asthma, and bronchiectasis as clinically relevant covariates. In a sensitivity analysis, blood eosinophil percentage was added to the primary model to examine the robustness of the association between CRS and mucus plugging. Given the limited number of mucus plug events, Firth’s penalized logistic regression, including CRS, asthma, bronchiectasis, and blood eosinophil percentage, was additionally performed to reduce potential small-sample bias.

For comparisons between two groups, Fisher’s exact test was used for categorical variables and the Mann–Whitney U test was used for continuous variables. Comparisons among the non-CRS, non-ECRS, and ECRS groups were performed using Fisher’s exact test for categorical variables and the Kruskal–Wallis test for continuous variables. When significant differences were detected, pairwise comparisons were performed using the corresponding two-group tests, with Holm’s method used to adjust for multiple comparisons.

Interobserver agreement for the presence of bronchiectasis and mucus plugs was assessed using Cohen’s kappa coefficient. Agreement for the BRICS and mucus plug scores was assessed using intraclass correlation coefficients (ICCs) based on a two-way random-effects model for absolute agreement with single measurements. The ICC for the BRICS was calculated among patients in whom bronchiectasis was identified by both readers.

All statistical analyses were conducted using EZR (Saitama Medical Center, Jichi Medical University, Saitama, Japan), a graphical user interface for R (version 4.5.2). A two-sided *p* value < 0.05 was considered statistically significant.

## 3. Results

### 3.1. Patient Characteristics According to CRS Status

The baseline characteristics of the study population are summarized in Table 1. A total of 97 patients were included, comprising 51 patients with CRS and 46 patients without CRS. The median age was 61.0 years (IQR, 47.5–69.0) in the CRS group and 52.0 years (IQR, 43.5–68.3) in the non-CRS group (*p* = 0.295). Women accounted for 31% and 41% of the CRS and non-CRS groups, respectively (*p* = 0.398). The proportions of never-smokers were 57% and 46%, respectively (*p* = 0.312), and median pack-years were 0.0 (IQR, 0.0–18.5) and 0.5 (IQR, 0.0–20.0), respectively (*p* = 0.352). The CRS group had a significantly higher white blood cell count than the non-CRS group (6700 [IQR, 5650–8050]/mm^3^ vs. 5450 [IQR, 4300–7200]/mm^3^, *p* = 0.005). Neutrophil percentages did not differ significantly between the CRS and non-CRS groups (55.7% [IQR, 51.6–61.4] vs. 58.2% [IQR, 50.0–62.7], *p* = 0.563), whereas eosinophil percentages were significantly higher in the CRS group (3.6% [IQR, 1.7–7.9] vs. 2.4% [IQR, 1.6–3.4], *p* = 0.022). Asthma was more frequent in the CRS group than in the non-CRS group (43% vs. 20%, *p* = 0.017), whereas allergic rhinitis was present in 51% and 48% of patients in the CRS and non-CRS groups, respectively (*p* = 0.840). Bronchiectasis was also more frequent in the CRS group (65% vs. 30%, *p* = 0.001), while BRICS among patients with bronchiectasis did not differ between the CRS and non-CRS groups (median, 1.0 [IQR, 1.0–2.5] vs. 1.0 [IQR, 1.0–2.0], *p* = 0.666). Among patients with CRS, nasal polyps were present in 18 patients (35%), the median Lund–Mackay score was 3.5 (IQR, 2.0–6.0), bilateral sinonasal involvement was observed in 35 patients (69%), and 18 patients (35%) were classified as having ECRS.

The indications for chest CT were respiratory symptoms in 36 patients (37.1%), evaluation or follow-up of respiratory diseases or abnormal chest findings in 15 (15.5%), non-respiratory symptoms or abnormal findings in 8 (8.2%), and evaluation, follow-up, or screening of other diseases in 38 (39.2%). The distribution of chest CT indications did not differ significantly between the CRS and non-CRS groups (*p* = 0.327). Paranasal sinus CT was performed for sinonasal symptom-related indications in 86 patients (88.7%) and for other indications in 11 (11.3%). Among the 46 patients without CRS, 35 (76.1%) underwent paranasal sinus CT for sinonasal symptom-related indications, whereas 11 (23.9%) underwent CT for other clinical indications. Individual indications for chest and paranasal sinus CT are shown in Supplementary Table S1. The median interval between paranasal sinus CT and chest HRCT was 145 days (IQR, 19–264). Chest HRCT was performed before paranasal sinus CT in two patients, with a maximum preceding interval of 16 days.

Interobserver agreement was high for both bronchiectasis (92.8% agreement; κ = 0.856) and the presence of mucus plugs (99.0% agreement; κ = 0.973). The ICC was 0.848 for the BRICS among the 47 patients in whom bronchiectasis was identified by both readers and 0.982 for the mucus plug score in the overall cohort.

### 3.2. Association Between CRS and Lower Airway Mucus Plugging

Mucus plugs were identified in 5 of 46 patients (10.9%) in the non-CRS group and 21 of 51 patients (41.2%) in the CRS group, indicating a significantly higher prevalence in patients with CRS (*p* = 0.001). The mucus plug score was also significantly higher in the CRS group than in the non-CRS group (median, 0.0 [IQR, 0.0–2.0] vs. 0.0 [IQR, 0.0–0.0], *p* = 0.002) (Table 2).

### 3.3. Logistic Regression Analyses for the Presence of Mucus Plugs

In univariable logistic regression analyses, CRS was significantly associated with the presence of mucus plugs (odds ratio [OR], 5.74; 95% confidence interval [CI], 1.94–17.0; *p* = 0.002). Asthma (OR, 3.71; 95% CI, 1.44–9.53; *p* = 0.007), bronchiectasis (OR, 3.26; 95% CI, 1.25–8.49; *p* = 0.016), and higher blood eosinophil percentage (OR per 1 percentage-point increase, 1.14; 95% CI, 1.03–1.26; *p* = 0.013) were also significantly associated with the presence of mucus plugs, whereas age, female sex, and never-smoking status were not significantly associated with the presence of mucus plugs. In the multivariable model including CRS, asthma, and bronchiectasis, CRS remained independently associated with the presence of mucus plugs (adjusted OR, 4.06; 95% CI, 1.30–12.7; *p* = 0.016), whereas asthma (adjusted OR, 2.54; 95% CI, 0.922–6.99; *p* = 0.071) and bronchiectasis (adjusted OR, 1.86; 95% CI, 0.651–5.34; *p* = 0.246) were not significantly associated. In the sensitivity analysis, additionally including blood eosinophil percentage, CRS remained significantly associated with the presence of mucus plugs (adjusted OR, 3.36; 95% CI, 1.05–10.8; *p* = 0.042), whereas blood eosinophil percentage was not independently associated with the presence of mucus plugs (adjusted OR per 1 percentage-point increase, 1.07; 95% CI, 0.973–1.18; *p* = 0.159) (Table 3). This association between CRS and the presence of mucus plugs was also confirmed using Firth’s penalized logistic regression including CRS, asthma, bronchiectasis, and blood eosinophil percentage (adjusted OR, 3.16; 95% CI, 1.08–10.36; *p* = 0.036) (Supplementary Table S2).

### 3.4. Association Between CRS Phenotypes and Mucus Plugging

Patients were classified into non-CRS (*n* = 46), non-ECRS (*n* = 33), and ECRS (*n* = 18) groups. The prevalence of mucus plugging differed significantly among the three groups (10.9%, 36.4%, and 50.0%, respectively; *p* = 0.001). In Holm-adjusted pairwise comparisons, the prevalence of mucus plugging was significantly higher in both the non-ECRS group (*p* = 0.023) and the ECRS group (*p* = 0.005) than in the non-CRS group, whereas no significant difference was observed between the non-ECRS and ECRS groups (*p* = 0.385) (Table 4).

Mucus plug scores also differed significantly among the three groups (*p* = 0.004), with median scores of 0.0 [IQR, 0.0–0.0], 0.0 [IQR, 0.0–2.0], and 0.5 [IQR, 0.0–2.8] in the non-CRS, non-ECRS, and ECRS groups, respectively. In Holm-adjusted pairwise comparisons, mucus plug scores were significantly higher in both the non-ECRS group (*p* = 0.025) and the ECRS group (*p* = 0.003) than in the non-CRS group, whereas no significant difference was observed between the two CRS phenotype groups (*p* = 0.314) (Table 4).

## 4. Discussion

In this retrospective single-center study, patients with CRS had a higher prevalence of lower airway mucus plugs and higher mucus plug scores than patients without CRS. Importantly, CRS remained associated with mucus plug presence after adjustment for asthma and bronchiectasis, and the association persisted after additional adjustment for blood eosinophil percentage, suggesting that this association was not fully explained by coexisting asthma or bronchiectasis. Both the non-ECRS and ECRS groups showed greater mucus plugging than the non-CRS group. However, the absence of a significant difference between the two CRS phenotypes should not be interpreted as evidence of equivalence because of the limited subgroup sample sizes.

The biological basis of this association remains uncertain, but several plausible pathways may be involved. The upper and lower airways form a continuous epithelial and immunological system, and sinonasal inflammation may influence the lower airway through shared inflammatory pathways and systemic trafficking of inflammatory cells [20]. In type 2 inflammation, interleukin (IL)-5 promotes eosinophilic inflammation, whereas IL-13 promotes goblet cell hyperplasia and mucin production, thereby creating conditions that favor mucus plug formation [21,22]. Activated eosinophils may further alter mucus properties through eosinophil peroxidase-mediated oxidative cross-linking of mucins and the release of extracellular DNA during eosinophil extracellular trap formation [10,23]. Non–type 2 pathways may also contribute to mucus plug formation through neutrophilic inflammation and proinflammatory cytokines that enhance airway mucin production [24]. Neutrophil extracellular traps may increase mucus viscoelasticity through the release of extracellular DNA, while neutrophil elastase may promote mucus hypersecretion [25,26]. Thus, although the inflammatory pathways differ, both eosinophilic and neutrophilic inflammation may promote mucus hypersecretion, altered mucus properties, and mucus retention. Impairment of mucociliary clearance may further contribute to mucus accumulation [27]. The finding that mucus plugging increased in both non-ECRS and ECRS is consistent with the possibility that multiple inflammatory pathways contribute to lower airway mucus plug formation in CRS.

From a clinical perspective, our findings identify mucus plugging as a potential radiological feature of the lower airways in patients with CRS. Previous studies have reported subclinical lower airway dysfunction in CRS, including latent obstructive changes in patients without asthma and impaired small airway function in non-asthmatic patients with CRS with nasal polyps (CRSwNP) [28,29]. A recent physiological study using spirometry, impulse oscillometry, and bronchial challenge testing further demonstrated lower airway dysfunction in patients with CRSwNP, including those without asthma [30]. The increased prevalence of mucus plugging observed in the present study raises the possibility that mucus plugging may contribute to the functional abnormalities reported in previous studies, although this relationship could not be assessed in the present study. The potential clinical significance of mucus plugging is further supported by observations in other chronic airway diseases. In asthma, lower airway mucus plugs are associated with airflow obstruction, respiratory symptom burden, and exacerbations [10,11,12]. In chronic obstructive pulmonary disease, greater mucus plug burden has been associated with adverse clinical outcomes, including mortality [31]. Prospective studies integrating chest imaging, respiratory symptoms, pulmonary function, and longitudinal outcomes will be needed to determine the functional and prognostic significance of mucus plugging in CRS.

Mucus plugging observed in the present study may also be interpreted as a radiological feature of the lower airways in sinobronchial syndrome (SBS), a condition characterized by the coexistence of chronic rhinosinusitis and chronic lower airway disease, including chronic bronchitis, bronchiolitis, and bronchiectasis. Impaired mucociliary clearance, mucus hypersecretion, and chronic airway inflammation in SBS may promote mucus plug formation. Consistent with this, increased sputum mucin concentrations and impaired lower airway mucociliary transport have been reported in patients with SBS [32,33].

Another important clinical question is whether lower airway mucus plugging associated with CRS is modifiable through treatment. Studies in asthma have demonstrated reductions in mucus plug burden following biologic therapy, with reductions in mucus plugging accompanying improvements in lung function or respiratory symptoms in some studies [19,34,35,36]. These findings indicate that mucus plugging can be a treatment-responsive feature in asthma. Although it remains unknown whether treatment directed at CRS can modify lower airway mucus plugging, a small study of patients with SBS reported improvements in lower airway symptoms and pulmonary function after endoscopic sinus surgery [37]. Longitudinal studies assessing mucus plugging before and after CRS treatment, followed by appropriately designed interventional studies, are needed to determine whether control of sinonasal disease can reduce lower airway mucus plugging and whether such changes contribute to improvements in clinical outcomes.

Several limitations should be acknowledged. First, the retrospective, single-center design introduces potential selection bias and precludes causal inference; therefore, the observed association does not establish that CRS causes lower airway mucus plugging. In addition, the study population was restricted to patients who underwent both chest HRCT and sinonasal evaluation and may therefore have been enriched for individuals with suspected or established lower airway disease, which may have introduced selection bias and limited generalizability to the broader CRS population. Second, the number of patients with mucus plugs was limited, resulting in a relatively low number of events per predictor parameter. In addition, residual confounding by age, sex, smoking exposure, other inflammatory characteristics, environmental exposures, and treatments cannot be excluded. Third, mucus plugs were assessed radiographically, and mucus composition, inflammatory mediators, airway microbiology, histopathology, and mucociliary function were not evaluated; therefore, the proposed biological mechanisms could not be directly examined. Fourth, pulmonary function and respiratory symptoms were not systematically assessed, preventing evaluation of the functional significance of mucus plugging. Fifth, the relatively small non-ECRS and ECRS subgroups limited the ability to detect phenotype-specific differences. In addition, ECRS was clinically defined using the JESREC score. Therefore, it remains unclear whether the observed association with mucus plugging applies to histopathologically confirmed ECRS. Sixth, detailed treatment information was not fully incorporated into the analysis, and potential treatment effects on mucus plugging could not be adequately assessed. Finally, the interval between paranasal sinus CT and chest HRCT was several months in some patients. Because mucus plugging may vary over time, this temporal discrepancy may have affected the observed association between CRS and mucus plugging.

## 5. Conclusions

CRS was associated with a higher prevalence and greater extent of lower airway mucus plugging on chest HRCT, and the association with mucus plug presence persisted after adjustment for asthma and bronchiectasis. Increased mucus plugging was observed in both ECRS and non-ECRS phenotypes. These findings suggest that mucus plugging may represent an underrecognized radiological feature of the lower airways in CRS. Further longitudinal studies are warranted to clarify the clinical implications of lower airway mucus plugging in patients with CRS.

## Figures and Tables

**Table 1:** Baseline characteristics of the study population.

Variable	CRS (*n* = 51)	Non-CRS (*n* = 46)	*p* Value
Age, years	61.0 (47.5–69.0)	52.0 (43.5–68.3)	0.295
Female, *n* (%)	16 (31)	19 (41)	0.398
Never-smoker, *n* (%)	29 (57)	21 (46)	0.312
Pack-years	0.0 (0.0–18.5)	0.5 (0.0–20.0)	0.352
White blood cell count, /mm^3^	6700 (5650–8050)	5450 (4300–7200)	**0.005**
Neutrophils, %	55.7 (51.6–61.4)	58.2 (50.0–62.7)	0.563
Eosinophils, %	3.6 (1.7–7.9)	2.4 (1.6–3.4)	**0.022**
Asthma, *n* (%)	22 (43)	9 (20)	**0.017**
Allergic rhinitis, *n* (%)	26 (51)	22 (48)	0.840
Bronchiectasis, *n* (%)	33 (65)	14 (30)	**0.001**
BRICS among patients with bronchiectasis	1.0 (1.0–2.5)	1.0 (1.0–2.0)	0.666
CRS characteristics			
Nasal polyps, *n* (%)	18 (35)	—	—
Lund–Mackay score	3.5 (2.0–6.0)	—	—
Bilateral sinonasal involvement, *n* (%)	35 (69)	—	—
ECRS, *n* (%)	18 (35)	—	—

Abbreviations: BRICS, Bronchiectasis Radiologically Indexed CT Score; CRS, chronic rhinosinusitis; ECRS, eosinophilic chronic rhinosinusitis. Data are presented as median (interquartile range) or n (%), unless otherwise indicated. Categorical variables were compared using Fisher’s exact test, and continuous variables were compared using the Mann–Whitney U test. Significant *p* values are shown in bold.

**Table 2:** Association between CRS and lower airway mucus plugging.

Variable	CRS (*n* = 51)	Non-CRS (*n* = 46)	*p* Value
Mucus plugging, *n* (%)	21 (41.2)	5 (10.9)	**0.001**
Mucus plug score, median (IQR)	0.0 (0.0–2.0)	0.0 (0.0–0.0)	**0.002**

Abbreviations: CRS, chronic rhinosinusitis; IQR, interquartile range. Categorical variables were compared using Fisher’s exact test, and continuous variables using the Mann–Whitney U test. Significant *p* values are shown in bold.

**Table 3:** Logistic regression analyses for factors associated with the presence of mucus plugs.

Variable	Univariable Analysis OR (95% CI), *p*	Primary Multivariable Model Adjusted OR (95% CI), *p*	Sensitivity Model Adjusted OR (95% CI), *p*
Age, per 1-year increase	1.01 (0.985–1.04), *p* = 0.359	—	—
Female sex	1.44 (0.572–3.61), *p* = 0.441	—	—
Never smoker	2.18 (0.856–5.53), *p* = 0.103	—	—
Asthma	**3.71 (1.44–9.53), *p* = 0.007**	2.54 (0.922–6.99), *p* = 0.071	1.79 (0.585–5.46), *p* = 0.308
Bronchiectasis	**3.26 (1.25–8.49), *p* = 0.016**	1.86 (0.651–5.34), *p* = 0.246	1.72 (0.591–5.00), *p* = 0.321
CRS	**5.74 (1.94–17.0), *p* = 0.002**	**4.06 (1.30–12.7), *p* = 0.016**	**3.36 (1.05–10.8), *p* = 0.042**
Blood eosinophils, per 1 percentage-point increase	**1.14 (1.03–1.26), *p* = 0.013**	—	1.07 (0.973–1.18), *p* = 0.159

Abbreviations: CI, confidence interval; CRS, chronic rhinosinusitis; OR, odds ratio. The primary multivariable model simultaneously included CRS, asthma, and bronchiectasis. The sensitivity model additionally included blood eosinophil percentage. Significant *p* values are shown in bold.

**Table 4:** Association between CRS phenotypes and lower airway mucus plugging.

Variable	Non-CRS (*n* = 46)	Non-ECRS (*n* = 33)	ECRS (*n* = 18)	Overall *p* Value
Mucus plugging, *n* (%)	5 (10.9)	12 (36.4)	9 (50.0)	**0.001**
Mucus plug score, median (IQR)	0.0 (0.0–0.0)	0.0 (0.0–2.0)	0.5 (0.0–2.8)	**0.004**
Pairwise comparisons, *p* value				
Variable	Non-ECRS vs. non-CRS	ECRS vs. non-CRS	ECRS vs. non-ECRS	
Mucus plugging	**0.023**	**0.005**	0.385	
Mucus plug score	**0.025**	**0.003**	0.314	

Abbreviations: CRS, chronic rhinosinusitis; ECRS, eosinophilic chronic rhinosinusitis; IQR, interquartile range. Overall comparisons were performed using Fisher’s exact test for categorical variables and the Kruskal–Wallis test for continuous variables. Pairwise comparisons were performed using Fisher’s exact test or the Mann–Whitney U test, as appropriate, with Holm adjustment for multiple comparisons. Significant *p* values are shown in bold.

## Data Availability

The data presented in this study are available from the corresponding author upon reasonable request, subject to institutional and ethical approval.

## Supplementary Materials

The following supporting information can be downloaded at: https://www.mdpi.com/article/10.3390/jcm15197609/s1, Table S1: Clinical indications for chest CT and paranasal sinus CT in individual patients; Table S2: Firth penalized logistic regression analysis for the presence of lower airway mucus plugs.
