# Supplementary material for: Chronic Rhinosinusitis Is Associated with Lower Airway Mucus Plugging
Source: J Clin Med. 2026 Sep 30;15(19):7609. doi: 10.3390/jcm15197609 (PMC13665754; doi:10.3390/jcm15197609)
Supplement: Supplementary file 1 [file jcm-15-07609-s001.zip › jcm-4537184-supplementary.pdf]

Indications were classified descriptively based on the documented reason for each examination.

Supplementary Table S1. Clinical indications for chest CT and paranasal sinus CT in individual patients.

No.	CRS status	Indication for chest CT	Chest CT category	Indication for paranasal sinus CT	Paranasal sinus CT category
1	CRS	Cough	Respiratory symptoms	Nasal discharge	Sinonasal symptom-related
2	CRS	Follow-up after surgery for tongue cancer	Evaluation/follow-up/screening of other diseases	Nasal obstruction and discharge	Sinonasal symptom-related
3	CRS	Cough and dyspnea	Respiratory symptoms	Nasal discharge and headache	Sinonasal symptom-related
4	CRS	Cough and sputum	Respiratory symptoms	Olfactory dysfunction, nasal obstruction, and discharge	Sinonasal symptom-related
5	CRS	Cough and sputum; evaluation of bronchiectasis	Respiratory disease or abnormal chest findings	Nasal discharge	Sinonasal symptom-related
6	CRS	Cough and sputum	Respiratory symptoms	Nasal discharge	Sinonasal symptom-related
7	CRS	Routine health checkup	Evaluation/follow-up/screening of other diseases	Facial pain	Sinonasal symptom-related
8	CRS	Cough	Respiratory symptoms	Nasal discharge	Sinonasal symptom-related
9	CRS	Cough	Respiratory symptoms	Nasal discharge	Sinonasal symptom-related
10	CRS	Follow-up of prostate cancer	Evaluation/follow-up/screening of other diseases	Facial pain	Sinonasal symptom-related
11	CRS	Systemic evaluation for abdominal pain	Non-respiratory symptoms or abnormal findings	Nasal obstruction and discharge	Sinonasal symptom-related
12	CRS	Abnormal chest radiograph	Respiratory disease or abnormal chest findings	Nasal discharge and obstruction	Sinonasal symptom-related
13	CRS	Evaluation of CRS	Evaluation/follow-up/screening of other diseases	Nasal discharge	Sinonasal symptom-related
14	CRS	Abnormal chest imaging finding	Respiratory disease or abnormal chest findings	Nasal obstruction	Sinonasal symptom-related
15	CRS	Evaluation of thyroid cancer	Evaluation/follow-up/screening of other diseases	Nasal discharge, nasal obstruction, and otitis media	Sinonasal symptom-related
16	CRS	Dyspnea and sputum	Respiratory symptoms	Olfactory dysfunction and nasal discharge	Sinonasal symptom-related
17	CRS	Systemic evaluation/staging of gastric cancer	Evaluation/follow-up/screening of other diseases	Headache	Sinonasal symptom-related
18	CRS	Follow-up of small pulmonary nodules	Respiratory disease or abnormal chest findings	Nasal discharge and obstruction	Sinonasal symptom-related
19	CRS	Evaluation of asthma	Respiratory disease or abnormal chest findings	Nasal discharge	Sinonasal symptom-related
20	CRS	Blood-streaked sputum and chest pain	Respiratory symptoms	Nasal discharge	Sinonasal symptom-related
21	CRS	Follow-up of lymphangioleiomyomatosis	Respiratory disease or abnormal chest findings	Nasal discharge	Sinonasal symptom-related
22	CRS	Follow-up after surgery for gastric cancer	Evaluation/follow-up/screening of other diseases	Nasal obstruction	Sinonasal symptom-related

No.	CRS status	Indication for chest CT	Chest CT category	Indication for paranasal sinus CT	Paranasal sinus CT category
23	CRS	Cough and sputum	Respiratory symptoms	Nasal discharge	Sinonasal symptom-related
24	CRS	Evaluation of abnormal pulmonary function test findings	Respiratory disease or abnormal chest findings	Olfactory dysfunction	Sinonasal symptom-related
25	CRS	Dyspnea and cough	Respiratory symptoms	Nasal discharge and olfactory dysfunction	Sinonasal symptom-related
26	CRS	Follow-up of enlarged obturator lymph nodes	Evaluation/follow-up/screening of other diseases	Olfactory dysfunction and nasal discharge	Sinonasal symptom-related
27	CRS	Follow-up of pulmonary embolism	Respiratory disease or abnormal chest findings	Nasal obstruction	Sinonasal symptom-related
28	CRS	Screening before antibody therapy	Evaluation/follow-up/screening of other diseases	Nasal discharge and obstruction	Sinonasal symptom-related
29	CRS	Preoperative evaluation for glioma	Evaluation/follow-up/screening of other diseases	Olfactory dysfunction, nasal discharge, and obstruction	Sinonasal symptom-related
30	CRS	Bronchiectasis follow-up; sputum	Respiratory disease or abnormal chest findings	Nasal discharge	Sinonasal symptom-related
31	CRS	Cough and dyspnea	Respiratory symptoms	Nasal discharge, olfactory dysfunction, and headache	Sinonasal symptom-related
32	CRS	Dyspnea	Respiratory symptoms	Olfactory dysfunction and nasal obstruction	Sinonasal symptom-related
33	CRS	Pre-biologic screening for psoriasis vulgaris	Evaluation/follow-up/screening of other diseases	Nasal obstruction and olfactory dysfunction	Sinonasal symptom-related
34	CRS	Bronchiectasis follow-up; sputum	Respiratory disease or abnormal chest findings	Nasal discharge	Sinonasal symptom-related
35	CRS	Follow-up after lymphoma remission	Evaluation/follow-up/screening of other diseases	Olfactory dysfunction	Sinonasal symptom-related
36	CRS	Follow-up of IgG4-related disease	Evaluation/follow-up/screening of other diseases	Nasal obstruction and olfactory dysfunction	Sinonasal symptom-related
37	CRS	Evaluation of pancreatic cancer	Evaluation/follow-up/screening of other diseases	Olfactory dysfunction	Sinonasal symptom-related
38	CRS	Evaluation of fever of unknown origin	Non-respiratory symptoms or abnormal findings	Nasal discharge and obstruction	Sinonasal symptom-related
39	CRS	Cough	Respiratory symptoms	Nasal discharge and obstruction	Sinonasal symptom-related
40	CRS	Cough and sputum	Respiratory symptoms	Nasal discharge and olfactory dysfunction	Sinonasal symptom-related
41	CRS	Evaluation of fever and abdominal pain	Non-respiratory symptoms or abnormal findings	Nasal discharge and obstruction	Sinonasal symptom-related
42	CRS	Follow-up of prostate cancer	Evaluation/follow-up/screening of other diseases	Nasal discharge	Sinonasal symptom-related
43	CRS	Evaluation of cerebral infarction	Evaluation/follow-up/screening of other diseases	Nasal discharge	Sinonasal symptom-related
44	CRS	Dyspnea	Respiratory symptoms	Nasal discharge	Sinonasal symptom-related
45	CRS	Cough	Respiratory symptoms	Nasal discharge	Sinonasal symptom-related

No.	CRS status	Indication for chest CT	Chest CT category	Indication for paranasal sinus CT	Paranasal sinus CT category
46	CRS	Evaluation of CRS	Evaluation/follow-up/screening of other diseases	Nasal discharge, olfactory dysfunction, and nasal obstruction	Sinonasal symptom-related
47	CRS	Preoperative evaluation for cholelithiasis	Evaluation/follow-up/screening of other diseases	Nasal discharge and obstruction	Sinonasal symptom-related
48	CRS	Cough	Respiratory symptoms	Nasal discharge	Sinonasal symptom-related
49	CRS	Blood-streaked sputum	Respiratory symptoms	Nasal discharge	Sinonasal symptom-related
50	CRS	Follow-up after surgery for thymic cancer	Evaluation/follow-up/screening of other diseases	Nasal discharge	Sinonasal symptom-related
51	CRS	Evaluation of an abnormal chest finding detected at health screening	Respiratory disease or abnormal chest findings	Facial pain	Sinonasal symptom-related
52	Non-CRS	Cough and blood-streaked sputum	Respiratory symptoms	Nasal discharge	Sinonasal symptom-related
53	Non-CRS	Evaluation of elevated β-D-glucan	Non-respiratory symptoms or abnormal findings	Evaluation of elevated β-D-glucan	Other indication
54	Non-CRS	Preoperative evaluation for cholelithiasis	Evaluation/follow-up/screening of other diseases	Facial pain	Sinonasal symptom-related
55	Non-CRS	Evaluation of an abnormal chest finding detected at health screening	Respiratory disease or abnormal chest findings	Evaluation of nasal crusting	Sinonasal symptom-related
56	Non-CRS	Evaluation of rib fracture	Evaluation/follow-up/screening of other diseases	Nasal discharge	Sinonasal symptom-related
57	Non-CRS	Cough	Respiratory symptoms	Evaluation of hearing loss	Other indication
58	Non-CRS	Cough	Respiratory symptoms	Nasal discharge and facial pain	Sinonasal symptom-related
59	Non-CRS	Follow-up of bladder cancer	Evaluation/follow-up/screening of other diseases	Evaluation of nasal septal deviation	Other indication
60	Non-CRS	Evaluation of pharyngeal cancer	Evaluation/follow-up/screening of other diseases	Nasal discharge	Sinonasal symptom-related
61	Non-CRS	Follow-up of lung cancer	Evaluation/follow-up/screening of other diseases	Maxillary pain	Sinonasal symptom-related
62	Non-CRS	Dyspnea	Respiratory symptoms	Nasal discharge	Sinonasal symptom-related
63	Non-CRS	Evaluation of Peutz–Jeghers syndrome	Evaluation/follow-up/screening of other diseases	Nasal discharge and obstruction	Sinonasal symptom-related
64	Non-CRS	Cough	Respiratory symptoms	Evaluation of asthma	Other indication
65	Non-CRS	Follow-up after treatment for maxillary sinus cancer	Evaluation/follow-up/screening of other diseases	Nasal discharge	Sinonasal symptom-related
66	Non-CRS	Evaluation of angina pectoris	Evaluation/follow-up/screening of other diseases	Nasal obstruction	Sinonasal symptom-related
67	Non-CRS	Follow-up of pancreatic cancer	Evaluation/follow-up/screening of other diseases	Facial pain	Sinonasal symptom-related

No.	CRS status	Indication for chest CT	Chest CT category	Indication for paranasal sinus CT	Paranasal sinus CT category
68	Non-CRS	Hemoptysis	Respiratory symptoms	Nasal discharge	Sinonasal symptom-related
69	Non-CRS	Follow-up of pulmonary nodule	Respiratory disease or abnormal chest findings	Nasal obstruction and cough	Sinonasal symptom-related
70	Non-CRS	Cough	Respiratory symptoms	Nasal discharge	Sinonasal symptom-related
71	Non-CRS	Follow-up of breast cancer	Evaluation/follow-up/screening of other diseases	Nasal discharge	Sinonasal symptom-related
72	Non-CRS	Follow-up of breast cancer	Evaluation/follow-up/screening of other diseases	Nasal discharge and obstruction	Sinonasal symptom-related
73	Non-CRS	Cough	Respiratory symptoms	Olfactory dysfunction	Sinonasal symptom-related
74	Non-CRS	Evaluation of elevated SCC antigen	Non-respiratory symptoms or abnormal findings	Evaluation of ocular pain	Other indication
75	Non-CRS	Cough	Respiratory symptoms	Nasal discharge and abnormal finding on brain screening	Sinonasal symptom-related
76	Non-CRS	Evaluation of hyperglycemia	Non-respiratory symptoms or abnormal findings	Olfactory dysfunction	Sinonasal symptom-related
77	Non-CRS	Cough and abnormal chest imaging finding	Respiratory disease or abnormal chest findings	Nasal discharge	Sinonasal symptom-related
78	Non-CRS	Evaluation of nasal vestibular cancer	Evaluation/follow-up/screening of other diseases	Evaluation of nasal vestibular cancer	Other indication
79	Non-CRS	Cough	Respiratory symptoms	Nasal discharge and headache	Sinonasal symptom-related
80	Non-CRS	Dyspnea	Respiratory symptoms	Nasal discharge	Sinonasal symptom-related
81	Non-CRS	Sensation of pharyngeal obstruction	Non-respiratory symptoms or abnormal findings	Nasal obstruction	Sinonasal symptom-related
82	Non-CRS	Follow-up of abdominal aortic aneurysm	Evaluation/follow-up/screening of other diseases	Evaluation of nasal crusting	Sinonasal symptom-related
83	Non-CRS	Follow-up after surgery for bladder cancer	Evaluation/follow-up/screening of other diseases	Olfactory dysfunction	Sinonasal symptom-related
84	Non-CRS	Cough	Respiratory symptoms	Nasal discharge	Sinonasal symptom-related
85	Non-CRS	Follow-up of pancreatic cancer	Evaluation/follow-up/screening of other diseases	Olfactory dysfunction	Sinonasal symptom-related
86	Non-CRS	Sputum and wheezing	Respiratory symptoms	Evaluation of asthma	Other indication
87	Non-CRS	Sputum and fever	Respiratory symptoms	Olfactory dysfunction and nasal discharge	Sinonasal symptom-related
88	Non-CRS	Follow-up of breast cancer	Evaluation/follow-up/screening of other diseases	Evaluation of nasal vestibular cyst	Other indication
89	Non-CRS	Follow-up of pulmonary nodule	Respiratory disease or abnormal chest findings	Follow-up of nasal septal deviation	Other indication
90	Non-CRS	Follow-up of cholangiocarcinoma	Evaluation/follow-up/screening of other diseases	Evaluation of taste disturbance	Other indication
91	Non-CRS	Evaluation of esophageal varices	Evaluation/follow-up/screening of other diseases	Nasal discharge and epistaxis	Sinonasal symptom-related
92	Non-CRS	Cough	Respiratory symptoms	Nasal discharge and facial pain	Sinonasal symptom-related

No.	CRS status	Indication for chest CT	Chest CT category	Indication for paranasal sinus CT	Paranasal sinus CT category
93	Non-CRS	Cough	Respiratory symptoms	Nasal obstruction	Sinonasal symptom-related
94	Non-CRS	Follow-up of lung cancer	Evaluation/follow-up/screening of other diseases	Nasal discharge	Sinonasal symptom-related
95	Non-CRS	Blood-streaked sputum, cough, and sputum	Respiratory symptoms	Evaluation of blood-streaked sputum	Other indication
96	Non-CRS	Cough	Respiratory symptoms	Nasal discharge	Sinonasal symptom-related
97	Non-CRS	Evaluation of epistaxis	Non-respiratory symptoms or abnormal findings	Epistaxis	Sinonasal symptom-related

Chest CT categories: respiratory symptoms, 36 (37.1%); respiratory disease or abnormal chest findings, 15 (15.5%); non-respiratory symptoms or abnormal findings, 8 (8.2%); evaluation/follow-up/screening of other diseases, 38 (39.2%). Paranasal sinus CT categories: sinonasal symptom-related, 86 (88.7%); other indication, 11 (11.3%). Abbreviations: CRS, chronic rhinosinusitis; CT, computed tomography.

Supplementary Table S2. Firth penalized logistic regression analysis for the presence of lower airway mucus plugs.

Variable	Adjusted OR	95% CI	p value
CRS	3.16	1.08–10.36	**0.036**
Asthma	1.82	0.61–5.29	0.279
Bronchiectasis	1.66	0.59–4.72	0.334
Blood eosinophils per 1 percentage-point increase	1.05	0.98–1.18	0.155

Abbreviations: CI, confidence interval; CRS, chronic rhinosinusitis; OR, odds ratio. Data were analyzed using Firth's penalized logistic regression with the presence of lower airway mucus plugs as the dependent variable. Chronic rhinosinusitis, asthma, bronchiectasis, and blood eosinophil percentage were simultaneously included as covariates. Significant p values are shown in bold.
